# Celecoxib as an Adjunctive Therapy in Patients With Bipolar Mania and Its Correlation With Interleukin 6: An Open Label Case Control Study

**DOI:** 10.1192/bjo.2025.10797

**Published:** 2025-06-20

**Authors:** Regina Rachel Khakha, D. Ram, Varun S. Mehta, K. K. Kshitij

**Affiliations:** 1 ESIC Medical College & Hospital, Faridabad, India; 2 Central Institute of Psychiatry, Ranchi, India

## Abstract

**Aims:** Recent research has focused on the inflammatory cascade as a key culprit in the aetiology of Bipolar disorder. We hypothesized that celecoxib, via its anti-inflammatory properties, may have a therapeutic role in mood disorder. The present study was a 4 weeks, open label case-control trial of celecoxib in patients of Bipolar mania as an adjunctive therapy to mood stabilizer and antipsychotic and to see its effect on IL-6 levels to objectively validate the improvement caused by celecoxib using this inflammatory marker.

**Methods:** This was a hospital-based, prospective, case-control study using purposive sampling. The study consisted of 50 participants of over 18 years of age, of which 25 received celecoxib (200 mg/day) adjunctive therapy to sodium valproate and a second generation antipsychotic while the other 25 received treatment as usual for 4 weeks. 25 healthy controls were also taken to measure and compare baseline serum Interleukin 6 levels. The Young Mania Rating Scale (YMRS), Brief Psychiatric Rating Scale (BPRS) and Clinical Global Impression – Severity scale (CGI-S) were used to assess severity of symptoms at baseline and at 4 weeks. The serum Interleukin 6 level was measured at baseline and at 4 weeks using an ELISA kit.

**Results:** The patients in each of the groups were comparable with respect to the socio-demographic, clinical characteristics and laboratory parameters at baseline. Interleukin 6 levels in the patient groups were significantly elevated when compared with healthy controls. Repeated measures ANOVA showed significant effect on treatment × time interaction on YMRS [F (1, 48) = 104.69, p<0.001] BPRS [F (1, 48) = 9.298, p = 0.004] and CGI-S [F(1, 48) = 65.774, p<0.0001] scores. YMRS, BPRS and CGI-S scores significantly decreased at 4 weeks in Bipolar patients receiving celecoxib in comparison to Bipolar patients receiving treatment as usual. There was a significant decrease in the serum Interleukin 6 (p<0.001) while on treatment with celecoxib adjunctive when compared with treatment as usual. The baseline Interleukin 6 levels correlated significantly with the improvement in symptoms (p<0.009) and the baseline score on YMRS scale was a predictor of the improvement.

**Conclusion:** This study found that celecoxib used as an adjunctive therapy with sodium valproate and antipsychotic in the treatment of Bipolar mania shows improvement in the manic and psychotic symptoms. It also significantly lowers Interleukin 6 levels of participants which were raised when compared with the healthy controls.

